# Iodine-Based Coordination Compounds: A Strategy Toward Antibiotic Potentiation

**DOI:** 10.3390/ijms27104292

**Published:** 2026-05-12

**Authors:** Daniil Shepilov, Dana Askarova, Anar Seisembekova, Seitzhan Turganbay, Ardak Jumagaziyeva, Tamara Bukeyeva, Gulnara Yuldasheva, Nurdaulet Temir, Lyudmila N. Ivanova, Natalya Zubenko, Sabina Kenesheva

**Affiliations:** 1 JSC Scientific Center for Anti-Infectious Drugs, Almaty 050060, Kazakhstan; shepilov2002@gmail.com (D.S.); askarova.dana08@gmail.com (D.A.); seysembekovaanar@gmail.com (A.S.); r_dawa@mail.ru (A.J.); bukeyeva_t@mail.ru (T.B.); yuldasheva57@rambler.ru (G.Y.); btnurdaulet@gmail.com (N.T.); lyudmila_69.69@mail.ru (L.N.I.); zubenkonatalie@gmail.com (N.Z.); silentium_n@bk.ru (S.K.); 2 Faculty of Natural Sciences, Kazakh National Women’s Teacher Training University, Almaty 050000, Kazakhstan; 3 Kazakh-British Technical University, School of Petroleum Engineering, “One Belt, One Road” Research Center, Almaty 050000, Kazakhstan

**Keywords:** iodine-containing compounds, active pharmaceutical substances, drug–drug synergy, novel antimicrobial adjuvants, resistance mitigation

## Abstract

Due to the increasing threat of antibiotic resistance and the emergence of new pathogenic strains, the development of effective combined therapeutic agents represents a crucial direction in the fight against infections. Within this study, several compounds were synthesized in which iodine is present in a coordination complex with antibiotics—sodium sulfadimidine and gentamicin sulfate. The physicochemical parameters of these compounds were investigated using capillary electrophoresis and UV spectroscopy, along with their cytotoxicity, antimicrobial, and antiviral activities. As a result of this work, two stable compounds, KC-246 and KC-248, were synthesized, demonstrating virus-inhibitory activity against herpes simplex virus and influenza A under extremely low cytotoxicity levels of 0.018–0.106 mg/mL. Additionally, they exhibited antimicrobial activity against representatives of the families *Staphylococcaceae*, *Pseudomonadaceae*, *Enterobacteriaceae*, *Enterococcaceae*, and yeast-like fungi. The minimum bactericidal concentrations (MBCs) ranged from 0.794 µg/mL to 0.198 µg/mL (KC-246) and from 2.093 µg/mL to 0.523 µg/mL (KC-248).

## 1. Introduction

Antibiotics have played a pivotal role in combating infectious diseases, substantially improving quality of life and extending human life expectancy. Despite these undeniable benefits, their extensive application in human medicine, agriculture, and veterinary practice has introduced significant environmental challenges. In particular, the continuous release of antibiotic residues into natural ecosystems—via wastewater effluents, livestock waste, and improper pharmaceutical disposal—has emerged as a critical driver of ecological imbalance and microbial adaptation.

Within this environmental context, the development of antimicrobial resistance (AMR), although an inherent evolutionary process, is being markedly accelerated. The excessive and often uncontrolled use of antibiotics, combined with inadequate waste management practices, creates selective pressure that favors resistant microbial populations. Consequently, antibiotic resistance has evolved from a localized clinical concern into one of the most pressing global public health threats of the 21st century. According to the United States Centers for Disease Control and Prevention (CDC), more than 2.8 million infections caused by antibiotic-resistant microorganisms are reported annually, resulting in over 35,000 deaths in the United States alone [1]. Beyond its clinical implications, AMR imposes a substantial economic burden; in the European Union, the costs associated with prolonged hospitalization and complex therapeutic regimens are estimated at approximately €1.5 billion per year [2].

The long-term consequences of this trend are particularly alarming. Projections by Naghavi, Mohsen et al. suggest that, in the absence of effective intervention strategies, annual mortality attributable to infections caused by resistant pathogens may exceed 10 million by 2050. This projected burden is comparable to global mortality from oncological diseases, highlighting not only the scale but also the urgency of the antimicrobial resistance crisis [3]. These forecasts underscore the critical need for innovative approaches that can preserve the efficacy of existing antimicrobial agents.

In response to this growing threat, current research efforts are increasingly focused on the development of antibiotic adjuvants and potentiators designed to enhance the activity of conventional drugs. Among these strategies, the synthesis of iodine-containing combined potentiators has attracted particular attention. Such compounds are capable of increasing microbial cell membrane permeability and suppressing the activity of antibiotic-degrading enzymes, thereby restoring or amplifying antibacterial efficacy against resistant strains [4,5].

At the molecular level, iodine exhibits distinctive physicochemical properties that allow it to penetrate microbial membranes and interact with intracellular targets. The antimicrobial activity of iodine-containing species, including elemental iodine, hypoiodous acid, and iodide ions, is primarily associated with their ability to interact with amino acids containing -NH_2_ groups (such as lysine, histidine, and arginine), as well as with nucleotides including adenine, cytosine, and guanine [6,7,8]. These interactions lead to the formation of N-iodinated derivatives, which disrupt essential cellular processes and compromise microbial viability. Notably, the capacity of iodine to access intracellular compartments is particularly advantageous in the treatment of infections with an intracellular life cycle, such as brucellosis, chlamydial infections, and viral hepatitis.

However, the practical application of iodine-based systems requires addressing their inherent chemical instability. Iodine and iodide species are susceptible to oxidative degradation under exposure to light, air, and moisture. To overcome this limitation, lithium iodide is employed as a stabilizing agent. Through its protective effect, lithium iodide inhibits iodine decomposition, thereby extending the shelf life of iodine-containing formulations and enhancing their stability and reliability in clinical practice [9,10,11].

The sulfonamide class of antibiotics (sodium sulfadimidine) exert a bacteriostatic effect by acting as structural analogues of para-aminobenzoic acid, competitively inhibiting the enzyme dihydropteroate synthase, a key catalyst in the microbial biosynthesis of folic acid necessary for purine and pyrimidine formation [12,13]. By interrupting folate synthesis, sulfonamides impede nucleic acid production and bacterial replication without directly lysing cells, resulting in growth inhibition across a broad spectrum of Gram-positive and Gram-negative organisms.

Aminoglycoside antibiotics (gentamicin) exert a bactericidal effect by binding to the 16S rRNA of the 30S ribosomal subunit, thereby impairing translational fidelity and inhibiting bacterial protein synthesis [14]. This interaction induces codon misreading and the production of aberrant proteins, which contributes to irreversible damage to bacterial cellular functions and loss of viability. Gentamicin is used primarily in the treatment of severe infections caused predominantly by aerobic Gram-negative microorganisms [15], and in selected combination regimens where synergistic antimicrobial activity is required.

The effectiveness of these stabilized iodine-containing systems can be further enhanced through co-administration with antibiotics of complementary molecular structures. Synergistic interactions between these agents can substantially increase antimicrobial activity, particularly against multidrug-resistant pathogens. Whereas monotherapy often results only in temporary growth inhibition, combination strategies exert multi-level pressure on microbial systems, providing a more robust approach capable of overcoming resistance mechanisms and achieving complete pathogen eradication.

## 2. Results

### 2.1. Computational Modeling of Molecular Interactions

**Figure 1:**
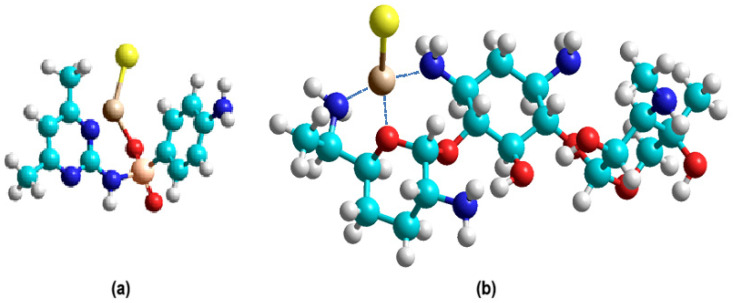
Suggested structures: (**a**) LiI + sulfadimidine; (**b**) LiI + gentamicin sulfate.

**Figure 2:**
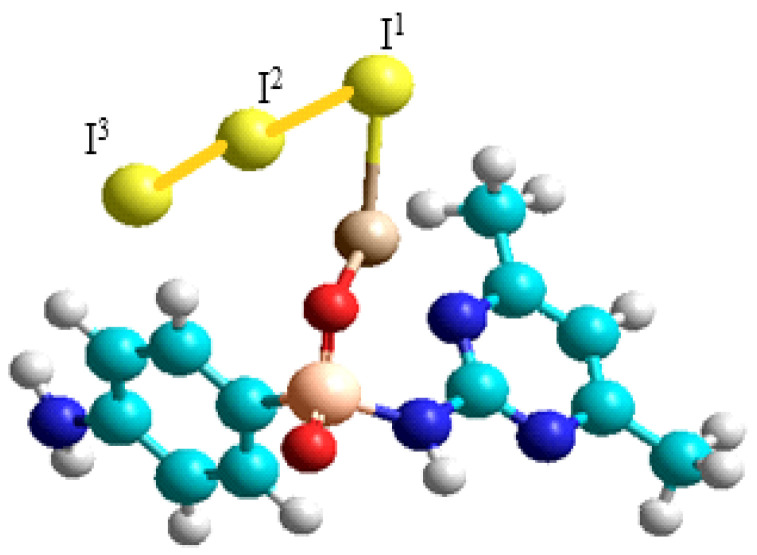
Suggested structure c: LiI_3_ + sodium sulfadimidine.

Within the framework of the quantum chemical investigation, an analysis of possible coordination interactions between the compound and triiodide and lithium iodide was performed. Three computational models of coordination complexes ab were constructed (Figure 1 and Figure 2), and their complexation energies were calculated to assess their thermodynamic stability (Table 1 and Table 2).

### 2.2. Synthesis and Physicochemical Characterization

For the synthesis of new active pharmaceutical ingredients (APIs) and the investigation of their physicochemical properties, the following stages were performed. A weighed portion of the antibiotics with an accuracy of ±0.001 g, was dissolved in 10 mL of distilled water under heating in a water bath at +40 °C with continuous stirring until complete dissolution. Solutions of lithium iodide (LiI) and crystalline iodine (I_2_) were prepared analogously in 10 mL of distilled water at room temperature.

The resulting lithium triiodide solution was added to the solution of the active substance with thorough stirring at RT for 5 min. The reaction mixture was kept in the dark at RT for 48 h to reach equilibrium. After the incubation period, the mixture was heated at +40 °C for 10 min, vacuum-filtered through a Schott filter, and transferred to a dark glass desiccator containing anhydrous calcium chloride to remove the bulk of water. The formed crystals were separated by vacuum filtration, washed twice with ethanol pre-cooled to 0 °C, and dried on an ashless filter. The dried crystals were weighed and placed into hermetically sealed glass vials for storage under refrigeration conditions.

During the varying of synthesis parameters (temperature, reaction time), it was established that the optimal conditions involve the use of an aqueous medium and temperatures up to 40 °C.

As a result of the synthesis, the compounds designated KC-246 (sodium sulfadimidine) and KC-248 (gentamicin sulfate) were obtained. The results of the physicochemical characterization are presented in Table 3.

The iodide ion concentration determined by capillary electrophoresis (CE) correlates well with the data obtained by titration, as shown in Table 4. In addition, quantitative determination of lithium cations was also performed using the CE method, and the corresponding results are presented in Table 5.

To confirm stability, the solubility of the samples was reassessed after 3 months (Table 6). The results showed that the solubility of KC-246 and KC-248 remained unchanged: both samples retained good solubility in water, as well as in DMSO and acetone at a ratio of 1 g/5 mL to 1 g/10 mL. These data confirm the stability of the chemical structure and the absence of significant degradative changes in the sample characteristics during the investigated period.

### 2.3. FT-IR (Fourier-Transform Infrared Spectroscopy) Spectroscopy

**Figure 3:**
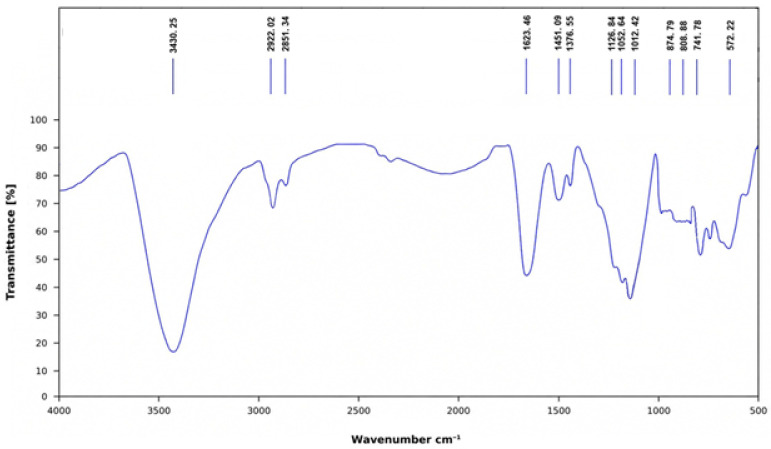
IR spectrum of KC-246 (sodium sulfadimidine).

The FT-IR spectrum of KC-246 (Figure 3) retains the characteristic features of sodium sulfadimidine, confirming preservation of the parent sulfonamide framework. A broad band at 3430.25 cm^−1^ corresponds to N–H stretching vibrations, while absorptions at 2922.02 and 2851.34 cm^−1^ are assigned to aliphatic C–H stretching. The band at 1623.46 cm^−1^ is attributed to C=N stretching of the heterocyclic ring, accompanied by aromatic C=C vibrations, with additional contributions at 1451.09 cm^−1^.

The sulfonyl group is clearly identified by strong bands at 1376.55 cm^−1^ (asymmetric) and 1126.84 cm^−1^ (symmetric SO_2_ stretching), indicating that the core functional group remains intact. Aromatic C–H out-of-plane bending vibrations are observed at 874.79 and 741.78 cm^−1^, while the band at 572.22 cm^−1^ corresponds to skeletal vibrations of the sulfonamide structure.

Notably, additional band at 1012.42 cm^−1^, along with a feature at 808 cm^−1^, are assigned to Li–O stretching vibrations, which are absent in the parent compound. These bands provide strong evidence of coordination involving lithium ions.

**Figure 4:**
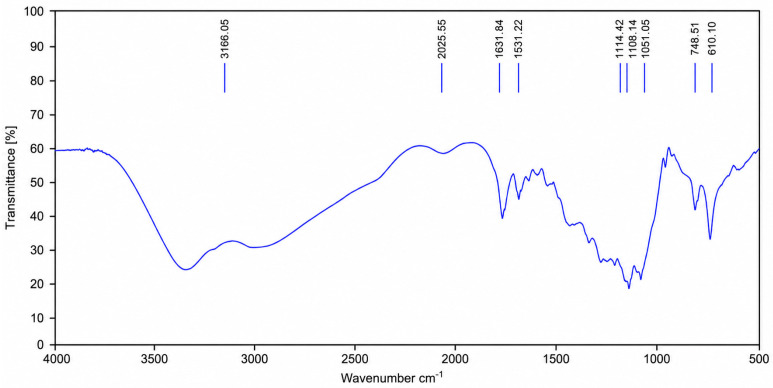
IR spectrum of KC-248 (gentamicin sulfate).

The FT-IR spectrum of KC-248 (Figure 4) retains the key vibrational features of gentamicin sulfate, confirming preservation of its aminoglycoside framework. The broad band at 3166.05 cm^−1^ corresponds to overlapping O–H and N–H stretching, while bands at 1631.84 and 1531.22 cm^−1^ are assigned to amine deformation vibrations. Intense absorptions at 1114.42 and 1051.05 cm^−1^ arise from C–O and C–N stretching modes characteristic of the carbohydrate-like structure, with additional skeletal features at 1108.14 and 748.51 cm^−1^.

Crucially, new bands are evident on 1051 cm^−1^ and 748.51 cm^−1^, absent in native gentamicin sulfate, and are assigned to Li–O stretching vibrations. Their appearance provides direct spectroscopic evidence of lithium coordination. Thus, the spectrum clearly demonstrates that the original functional groups of gentamicin are preserved, while the emergence of Li–O modes confirm the formation of the coordination compound KC-248.

### 2.4. UV Spectroscopic Methods of Analysis

**Figure 5:**
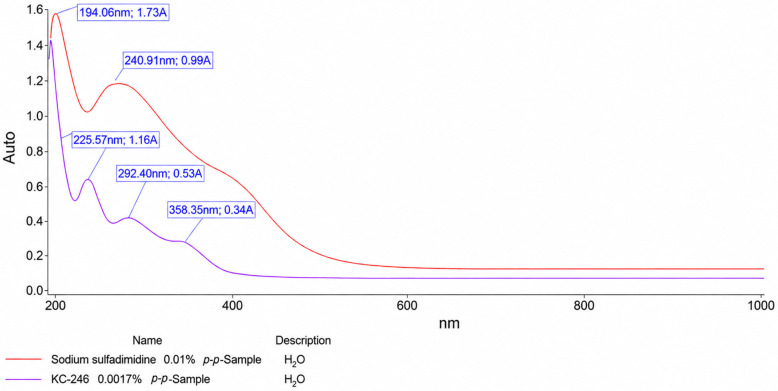
UV spectra of KC-246 0.0017% and sodium sulfadimidine 0.01%.

**Figure 6:**
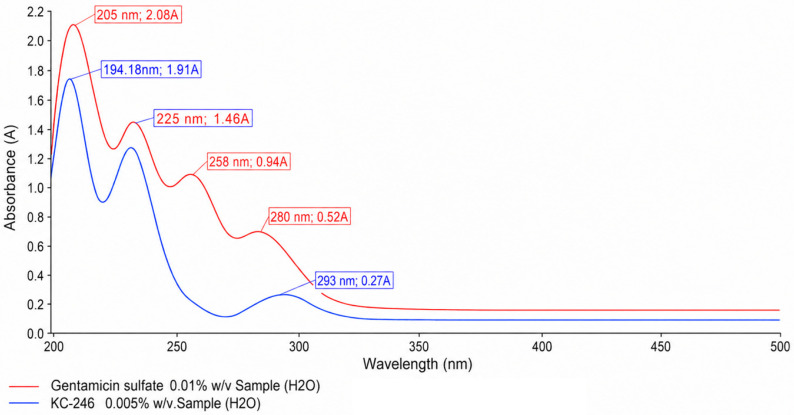
UV spectra of KC-248 (0.005%) and gentamicin sulfate (0.01%).

The obtained spectral characteristics, with indication of the transitions, are presented in Table 7, Figure 5 and Figure 6.

### 2.5. In Vitro Safety Testing

The cytotoxicity of the synthesized APSs KC-246 and KC-248 was investigated on human peripheral blood mononuclear cell (PBMC) cultures, as well as on MDCK and RD cells under in vitro conditions. The CC_50_ values were determined by the nonlinear regression method using GraphPad Prism 6 software based on the full concentration range.

To determine the cytotoxic concentration of KC-248, PBMCs (1 × 10^5^ cells/well) were seeded into 96-well plates with the addition of the substance at concentrations of 0.02–5.0 mg/mL, where 5.0 mg/mL was used as the maximum dose. The control group consisted of cells with the addition of RPMI-1640 medium.

For KC-246, the investigated concentrations were 0.002–0.500 mg/mL; the control group consisted of cells with 0.1% DMSO, since it served as the solvent. Each concentration was added in triplicate.

Incubation of PBMCs was carried out for 48 h at 37 °C, 5% CO_2_, and 95% humidity, after which the cytotoxic effect was assessed by the MTT assay based on cell viability.

**Figure 7:**
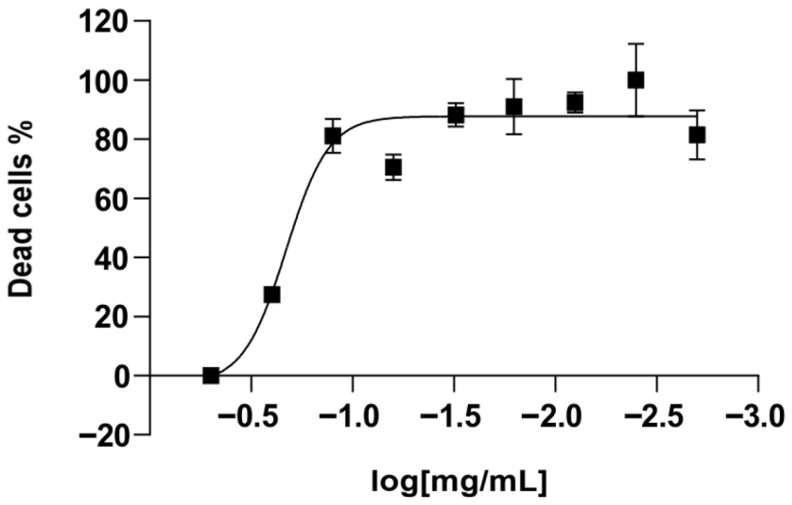
Dose–response curve for compound KC-246 on PBMC culture.

**Figure 8:**
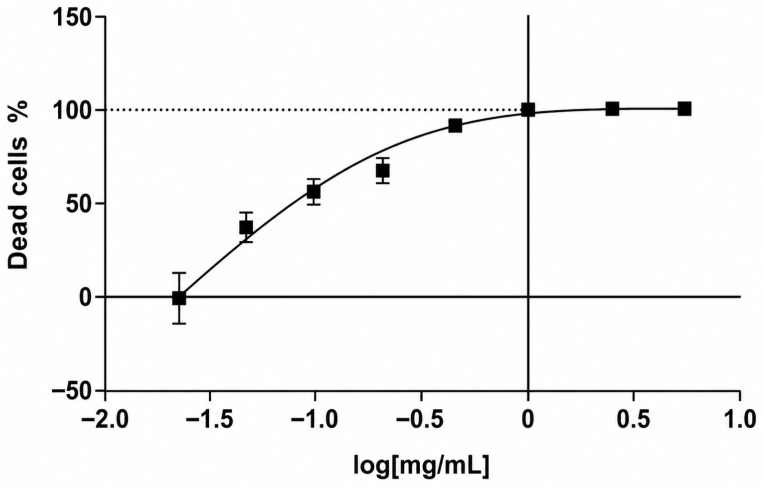
Dose–response curve for compound KC-248 on PBMC culture.

For KC-246, 48 h exposure at concentrations of 0.25–0.5 mg/mL caused a cytotoxic effect exceeding the permissible limit of (55 ± 5) %. The calculated CC_50_ value was 0.179 mg/mL (95% confidence interval: 0.149–0.214). The compound KC-248 exhibited cytotoxicity at concentrations of 1.25–5.0 mg/mL. The CC_50_ value was 0.425 mg/mL (95% confidence interval: 0.351–0.514) (Table 8) (Figure 7 and Figure 8).

The maximum non-toxic concentration for KC-246 is 0.045 mg/mL, and for KC-248 it is 0.106 mg/mL.

The cytotoxic effects of the newly synthesized compounds KC-246 and KC-248 were evaluated in MDCK and RD cell culture models following 72 h of incubation, and the corresponding results are presented in Table 9. 

MDCK and RD cell cultures were seeded into 96-well plates at a concentration of 2 × 10^5^ cells/mL. Cultivation was carried out at 37 °C and 5% CO_2_.

To assess the cytotoxicity of KC-246, the test substance was administered once at the following concentrations: 1.0 mg/mL, 0.5 mg/mL, 0.25 mg/mL, 0.125 mg/mL, 0.063 mg/mL, 0.031 mg/mL, 0.016 mg/mL, 0.008 mg/mL. For KC-248, the concentrations were: 20.0 mg/mL, 10.0 mg/mL, 5.0 mg/mL, 2.5 mg/mL, 1.25 mg/mL, 2.0 mg/mL, 1.0 mg/mL, 0.5 mg/mL, 0.25 mg/mL, 0.125 mg/mL, 0.063 mg/mL, 0.031 mg/mL, 0.016 mg/mL.

The exposure lasted 72 h. Upon completion of incubation, the optical density in the wells was measured using a Tecan Sunrise RC.4 plate reader (ecan Austria GmbH, Grödig/Salzburg, Austria) at wavelengths of 540 nm (main filter) and 620 nm (reference filter).

### 2.6. Antimicrobial Activity Screening

The antimicrobial activity of KC-246 and KC-248 substances was investigated against reference and clinical strains. Testing was performed by the method of two-fold serial dilutions in physiological saline.

During the studies, the minimum bactericidal concentrations (MBCs) of the samples were determined. These values were taken as the lowest concentration of the sample in dilution that completely suppressed the growth of the test microorganism on a Petri dish after inoculation onto the corresponding medium. The obtained study data are presented in Table 10.

### 2.7. Virus-Inhibitory Activity Screening

The antiviral activity of KC-246 and KC-248 substances was investigated on MDCK and RD cell cultures, based on the calculated CC_50_ values. The study was carried out in vitro to assess the effect on influenza A/Swine/Iowa/15/30 (H1N1) virus and herpes simplex virus type 1 (HSV-1), strain “Victory”, using a virus-inhibitory scheme of substance administration.

The virus was used at a dose of 100 ID/0.2 mL, and untreated virus was used as the control. The efficacy of the substances was assessed by comparing the levels of inhibition of viral activity in the experimental and control groups. The study results are presented in Table 11 and Table 12.

## 3. Discussion

In the current study, the formation energies of LiI complexes with sulfadimidine (a) and gentamicin sulfate (b) (Figure 1) were calculated using the DFT/B3PW91/6-31G** quantum chemical method [16,17] (Table 1).

The calculated data indicate that the stability of the complexes depends on the type of metal ion and the donor activity of the heteroatoms of the active substance. The most stable complexes are formed with LiI. The I_2_ bond dissociation energy in structure c (Figure 2) was calculated using the DFT/B3PW91/6-31G** quantum chemical method (Table 2). The calculated interaction energy of I_2_ with a potential protein binding site (ΔE = –2.23 kcal/mol) is significantly lower than the interaction energy of I_2_ in structure c.

Computational results suggest that complex c possesses the capability to deliver molecular iodine to nucleotide triplets of bacterial DNA.

The stability of the obtained compounds over a period of 3 months was evaluated according to the following parameters: pH, melting point, iodine content, and iodide content. Since the synthesized coordination compounds are considered as potential medicinal agents, their stability is a critical factor for further pharmaceutical investigations.

The solubility of samples KC-246 and KC-248 was examined. Both samples demonstrated high solubility in water as well as in aprotic organic solvents such as dimethyl sulfoxide (DMSO) and acetone at a ratio of 1 g/5 mL to 1 g/10 mL. This high solubility may be attributed to structural modifications that enhance solvent interactions while reducing intermolecular forces within the polymeric matrix.

The FT-IR analysis of both KC-246 and KC-248 (Figure 3 and Figure 4) demonstrates that the structural integrity of the parent pharmaceutical molecules is maintained following complex formation, while simultaneously providing evidence for successful coordination with lithium ions. In KC-246, the preservation of the sulfonamide-associated N–H, SO_2_, heterocyclic C=N, and aromatic vibrational bands confirms that sodium sulfadimidine retains its fundamental molecular architecture. The emergence of additional Li–O stretching bands at 1012.42 and 808 cm^−1^, absent in the precursor compound, strongly indicates the incorporation of lithium into the coordination environment.

Similarly, in KC-248, the characteristic aminoglycoside spectral profile of gentamicin sulfate remains largely unchanged, with preservation of hydroxyl, amino, and carbohydrate-associated vibrational modes. The appearance of new Li–O-associated absorptions at 1051 and 748.51 cm^−1^ provides clear confirmation of lithium coordination without disrupting the principal antibiotic scaffold.

Collectively, these findings indicate that the synthesis strategy successfully generates coordination compounds while preserving the biologically relevant structural motifs of the parent antimicrobial agents. The coexistence of preserved functional group signatures and newly formed Li–O vibrations support the hypothesis that lithium ions are incorporated through coordination interactions rather than destructive chemical modification.

Figure 5 and Figure 6 present the UV spectra of the KC-246 and KC-248 complexes together with their parent antibiotic components, sodium sulfadimidine and gentamicin sulfate. Both parent antibiotics exhibit characteristic absorption bands in the ultraviolet region, which are attributed to intrinsic π → π* and *n* → π* electronic transitions of their organic chromophores. Sodium sulfadimidine displays dominant maxima at 194.06 nm and 240.91 nm, whereas gentamicin sulfate demonstrates absorption bands at 205, 225, 258, and 280 nm. Following complex formation, both systems retain an intense short-wavelength peak near 194 nm (194.06 nm for KC-246 and 194.18 nm for KC-248), which, according to Edis et al., is a characteristic spectral marker of iodide ions (I^−^). This indicates that iodide represents the predominant iodine species in both synthesized systems.

Additional absorption bands around 225 nm observed in both complexes are most plausibly associated with ligand-to-iodine charge-transfer transitions, as well as alterations in the electronic states of the antibiotic molecules resulting from iodine coordination.

The most significant common spectral feature of both KC-246 and KC-248 is the emergence of new absorption bands in the 292–293 nm region, which correspond to the formation of triiodide (I_3_^−^) structures. In KC-246, an additional pronounced maximum is observed at 358.35 nm, whereas this band is less intense in KC-248, which may indicate a lower degree of triiodide stabilization. Nevertheless, the presence of absorption bands at ~292–293 nm in both complexes confirms that iodine incorporation is accompanied by the formation of triiodide moieties analogous to the “smart triiodides” described by Edis and Bloukh, where characteristic absorption maxima are typically reported near ~291 and ~359 nm.

Thus, both systems should not be regarded as simple mixtures of antibiotics with iodine, but rather as complex supramolecular assemblies in which a dynamic equilibrium exists between iodide, molecular iodine, and triiodide species. KC-246 demonstrates a more pronounced polyiodide organization with clearly stabilized triiodide structures, whereas KC-248 likely contains less stabilized polyiodide forms. However, in both cases, the formation of such structures indicates the successful transformation of the parent antibiotics into novel iodine-coordinated systems.

**Figure 9:**
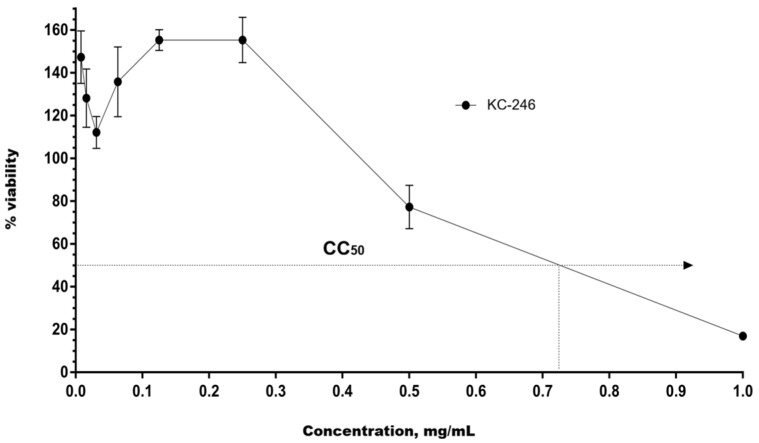
Cytotoxic effect of compound KC-246 on MDCK cell culture.

**Figure 10:**
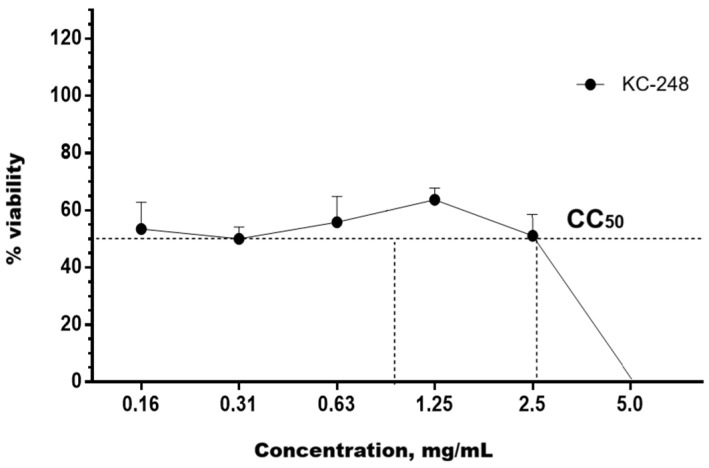
Cytotoxic effect of compound KC-248 on MDCK cell culture.

**Figure 11:**
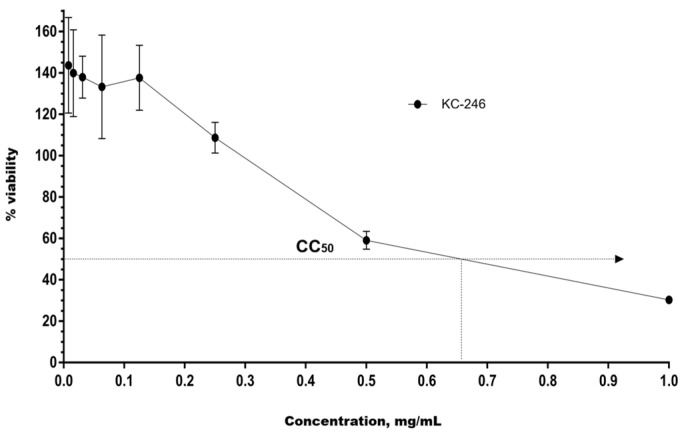
Cytotoxic effect of compound KC-246 on RD cell culture.

**Figure 12:**
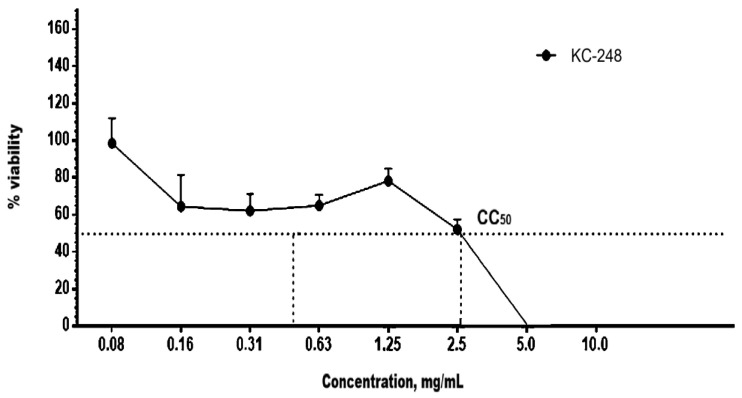
Cytotoxic effect of compound KC-248 on RD cell culture.

The study results confirmed the CC_50_ values for KC-246 and KC-248 in MDCK (Figure 9 and Figure 10) and RD (Figure 11 and Figure 12) cell cultures after 72 h of incubation, which indicates the cytotoxic effect of these newly synthesized potentiators.

From the data of Table 10, it was established that KC-246 possesses antimicrobial activity against all test strains. The coordination compound KC-246 exhibits identical activity against representatives of the Enterobacteriaceae family (*E. coli* ATCC 8739, *E. coli* ATCC 2523, *P. aeruginosa* TA2, *Kl. pneumoniae* ATCC 2524, *Kl. pneumoniae* ATCC 10031, *S. enterica* ATCC 14028) and a representative of the enterococci family (*Ent. hirae* ATCC 10541); the MBC was 0.794 μg/mL. Against *S. aureus* ATCC 33591, *Ent. faecalis* ATCC 51575, *P. aeruginosa* ATCC 9027, and *S. enterica* ATCC 35988, the minimum bactericidal concentration was 0.397 μg/mL. The MBC against Staphylococcus aureus (*S. aureus* ATCC 6538) was 0.198 μg/mL. Against the yeast-like fungi *C. albicans* ATCC 10231 (reference strain) and *C. albicans* SCAID PHPX 1-2019 (clinical strain), the minimum bactericidal concentration (MBC) was 0.794 μg/mL.

The coordination compound KC-248 also possesses antimicrobial activity against all tested microorganisms. Thus, it was established that against representatives of the genus Escherichia (*E. coli* ATCC 8739, *E. coli* ATCC 2523), *S. aureus* ATCC 33591, *S. enterica* ATCC 35988, *Kl. pneumoniae* ATCC 10031, and *Ent. hirae* ATCC 10541, the minimum bactericidal concentration was 0.787 μg/mL. Against representatives of Gram-positive cocci, namely staphylococci (*S. aureus* ATCC 6538) and enterococci (*Ent. faecalis* ATCC 51575), as well as *S. enterica* ATCC 14028, *P. aeruginosa* TA2, and yeast-like fungi (*C. albicans* ATCC 10231, *C. albicans* SCAID PHPX 1-2019), the MBC was 3.149 μg/mL. The minimum bactericidal concentration against representatives of the Enterobacteriaceae family, namely *Kl. pneumoniae* ATCC 2524, was recorded at 0.393 μg/mL, whereas against *P. aeruginosa* ATCC 9027 it was 1.574 μg/mL.

Thus, it was experimentally established that the coordination compounds KC-246 and KC-248 possess antimicrobial activity against Gram-positive, Gram-negative microorganisms and yeast-like fungi.

The study of the antiviral activity of the coordination compounds against influenza A/Swine/Iowa/15/30 (H1N1) virus showed that the substance KC-246, at concentrations of 0.365 mg/mL and 0.183 mg/mL, completely suppresses viral replication, providing a 100% inhibitory effect. Likewise, the substance KC-248 demonstrates a 100% reduction in influenza A virus replication at a concentration of 1.275 mg/mL.

In the study of antiviral activity against herpes simplex virus type 1, strain “Victory”, it was established that KC-246, at concentrations of 0.330 mg/mL and 0.165 mg/mL, completely suppresses viral replication, achieving a 100% inhibitory effect. At the same time, the substance KC-248 exhibits 100% antiviral activity against herpes simplex virus type 1, strain “Victory”, at a concentration of 1.300.

Thus, KC-246 and KC-248 demonstrated pronounced virus-inhibitory activity against both viruses.

## 4. Materials and Methods

### 4.1. Reagents

Glass laboratory ware was used throughout the study, including beakers of 50 and 100 mL, a 100 mL volumetric flask, and graduated cylinders of 10 and 25 mL (Duran, Hradec Králové, Czech Republic). Filter paper “Red Ribbon” (Hahnemühle FineArt GmbH, Dassel, Germany) and a Schott filter (Simax, Sázava, Czech Republic) were employed for filtration.

Analytical grade reagents were obtained from commercial suppliers and used without further purification: lithium iodide (Sigma-Aldrich, Burlington, MA, USA, ≥99%), iodine (Labpharm, JSC “Troitsky Iodine Plant”, Krasnodar, Russia, ≥98%), sodium sulfadimidine, and gentamicin sulfate (Sigma-Aldrich, Burlington, MA, USA, ≥99%).

### 4.2. Test Systems

The bacterial and fungal test strains were obtained from the American Type Culture Collection (ATCC) through the official distributor for Central Asia LGC Standards (Poland). Both reference (museum) and clinical strains, including sensitive and multidrug-resistant variants, were employed as follows:

1. *Staphylococcus aureus* ATCC 6538-P (museum, sensitive strain)
2. *Staphylococcus aureus* ATCC 333591 (museum, multidrug-resistant strain)
3. *Escherichia coli* ATCC 8739 (museum, sensitive strain)
4. *Escherichia coli* ATCC BAA-2523 (museum, multidrug-resistant strain)
5. *Pseudomonas aeruginosa* ATCC 9027 (museum, sensitive strain)
6. *Pseudomonas aeruginosa* TA2 (clinical, multidrug-resistant strain)
7. *Klebsiella pneumoniae* ATCC 10031 (museum, sensitive strain)
8. *Klebsiella pneumoniae* ATCC 2524 (museum, multidrug-resistant strain)
9. *Salmonella enterica* ATCC 14028 (museum, sensitive strain)
10. *Salmonella enterica* ATCC 35988 (museum, multidrug-resistant strain)
11. *Enterococcus hirae* ATCC 10541 (museum, sensitive strain)
12. *Enterococcus faecalis* ATCC 51575 (museum, multidrug-resistant strain)
13. *Candida albicans* ATCC 10231 (museum, sensitive strain)
14. *Candida albicans* SCID PHRX 1-2019 (clinical, multidrug-resistant strain)

Virus strains—Influenza A virus A/Swine/Iowa/15/30 (H1N1) and Herpes Simplex Virus type 1 (strain “Victory”), obtained from the Laboratory of Viral Ecology, Institute of Virology and Microbiology, Committee of Science, MES RK.

Cell cultures—MDCK cells (Madin–Darby Canine Kidney), obtained from the Laboratory of Cellular Biotechnology, Research Institute for Biological Safety Problems, National Center for Biotechnology, MES RK, and RD cells (human embryonal rhabdomyosarcoma), obtained from the cell culture collection of the National Center of Infectious and Parasitic Diseases, Sofia, Republic of Bulgaria.

Human peripheral blood mononuclear cells (PBMCs)—isolated from healthy donors (without acute or severe chronic diseases) of both sexes.

### 4.3. Preparation of the APSs

Accurately weighed samples of sodium sulfadimidine and flunixin meglumine (±0.001 g) were dissolved in 10 mL of distilled water by heating in a water bath at 40 °C with continuous stirring until complete dissolution. Solutions of lithium iodide (LiI) and crystalline iodine (I_2_) were prepared similarly in 10 mL of distilled water at room temperature (RT). The resulting lithium triiodide solution was added to the active substance solution, and the mixture was stirred thoroughly at RT for 5 min. The reaction mixture was then kept in the dark at RT for 48 h to reach equilibrium.

After the incubation period, the mixture was heated at 40 °C for 10 min, vacuum-filtered through a Schott filter, and transferred to a dark glass desiccator containing anhydrous calcium chloride to remove the majority of water. The resulting crystals were collected by vacuum filtration, washed twice with ethanol precooled to 0 °C, and dried on an ashless filter. The dried crystals were weighed and stored in airtight glass vials under refrigeration.

### 4.4. Quantum-Chemical Calculations

The DFT/B3PW91/6-31G** method used for quantum chemical calculations is based on density functional theory (DFT) employing the hybrid B3PW91 functional, which combines Hartree–Fock exchange–correlation effects with gradient-corrected correlation, providing high accuracy in predicting electronic structure. The 6-31G** basis set includes polarization functions, allowing an adequate description of the spatial distribution of electron density, as well as inductive and dispersion interactions. This method is particularly suitable for calculations of antibiotics in complex with lithium iodide, as it accurately captures ion–dipole interactions, specific solvation effects, charge delocalization, and hydrogen-bond formation. All computations were carried out on a Fujitsu PRIMERGY BX920 S1 supercomputer with a peak performance of 10.9 TFLOPS.

### 4.5. FT-IR Spectroscopy

The samples were processed without prior preparation via placing directly onto the ATR (attenuated total reflectance) diamond crystal and pressing to ensure optimal contact, their FT-IR spectra were collected using an FT-IR spectrometer (Bruker Alpha II FT-IR spectrometer, Bruker Optics GmbH, Ettlingen, Germany). The test conditions were as follows: wavenumber range, 4000–400 cm^−1^; scan number, 32; and resolution, 4 cm^−1^.

### 4.6. UV-Vis Spectroscopy

Ultraviolet–visible (UV–Vis) absorption spectra of the investigated systems were obtained using a double-beam Lambda 35 spectrophotometer (PerkinElmer, Waltham, MA, USA), equipped with a monochromator operating over the 190–1100 nm spectral range. Spectral acquisition was carried out in the 190–500 nm wavelength interval under continuous scanning (SCAN mode). Measurements were performed in 1.0 cm path length quartz cuvettes at room temperature.

### 4.7. Capillary Electrophoresis (CE)

The analyses were conducted using an Agilent 1600 capillary electrophoresis system (Agilent Technologies, Santa Clara, CA, USA) equipped with a diode-array detector (DAD). Analytical solutions were prepared at analyte concentrations ranging from 5 to 200 mg/L and filtered through 0.2 µm membrane filters prior to injection.

Qualitative identification was performed by comparing the migration times of the analyte ions with those of reference standards. Quantitative analysis was carried out by comparison of peak areas with those obtained for certified reference standards (CRS).

Cation determination was performed according to a validated internal analytical protocol using an uncoated fused-silica capillary (50 µm internal diameter, total length 56 cm). The background electrolyte (BGE) was adjusted to pH 3.2. Electrophoretic separation was conducted at 25 °C under normal polarity with an applied voltage of +30 kV. UV detection was performed at 310 and 215 nm.

Iodide ions were analyzed using a separate validated internal protocol under the following conditions: uncoated fused-silica capillary (50 µm i.d., 56 cm total length), BGE pH 9.3, reversed polarity (−30 kV), and UV detection at 226 nm. Both analytical procedures were validated in compliance with the requirements of the State Pharmacopoeia of the Republic of Kazakhstan and relevant literature sources [18,19,20].

### 4.8. Quantitive Analysis of Iodine and Iodide

The quantitative determination of molecular iodine in the obtained complexes was performed by iodometric titration with sodium thiosulfate in the presence of starch indicator.

An accurately weighed sample (0.1–0.3 g) was transferred into a 50 mL beaker, dissolved in 20 mL of purified water, and titrated with 0.05 M sodium thiosulfate solution until the brown coloration disappeared. Subsequently, 1–2 drops of starch solution were added, and titration was continued until complete decolorization of the solution (endpoint).

The iodine content (C_I2_) per 1 kg of the preparation, expressed in grams, was calculated using Equation (1):

C_I2_ = V_1_ × K_1_ × 12.69/m, (1)

where:

V_1_—volume of sodium thiosulfate solution consumed for titration, mL;
K_1_—correction factor for the molar concentration of 0.05 M sodium thiosulfate solution (K_1_ = 0.5);
m—sample mass, g.

According to the State Pharmacopoeia of the Republic of Kazakhstan, 1 mL of 0.1 M sodium thiosulfate corresponds to 12.69 g of iodine.

Potassium iodide in the obtained complexes was quantified by an electrochemical method according to a validated in-house procedure. After completion of iodine titration, 5 mL of 0.1 N nitric acid was added to the resulting solution, followed by potentiometric titration with 0.05 M silver nitrate solution.

Potentiometric measurements were performed using a combined platinum electrode with an integrated silver/silver chloride reference electrode. The equivalence point was determined from the titration curve (electrode potential versus volume of AgNO_3_ solution) as the midpoint of the potential jump.

The potassium iodide content (C_KI_) per 1 kg of the preparation, expressed in grams, was calculated using Equation (2):

C_KI_ = (V_2_ × K_2_ − V_1_ × K_1_) × 16.60/m, (2)

where:

V_2_—volume of silver nitrate solution consumed for sample titration, mL;
K_2_—correction factor for the molar concentration of 0.05 M silver nitrate solution (K_2_ = 0.5);
V_1_—volume of sodium thiosulfate solution used for iodine titration, mL;
K_1_—correction factor for the molar concentration of 0.05 M sodium thiosulfate solution (K_1_ = 0.5);
m—sample mass, g.

According to the State Pharmacopoeia of the Republic of Kazakhstan, 1 mL of 0.1 M silver nitrate corresponds to 16.60 g of potassium iodide.

### 4.9. Serial Dilution Method

The testing procedure and evaluation of antimicrobial activity were performed using the broth serial dilution method in accordance with the guidelines of the Clinical and Laboratory Standards Institute (CLSI) [21,22,23,24,25]. All experiments were carried out in triplicate to ensure reproducibility of the results.

### 4.10. Endpoint Dilution Assay for Infectious Titer Determination

The infectious titer of influenza A virus was determined by the limiting dilution assay in MDCK cell culture (MDCK). The presence of virus was confirmed by the hemagglutination assay performed according to the standard protocol using a 0.75% suspension of human erythrocytes (blood group I, O) [26,27].

The infectious titer of herpes simplex virus was determined by an analogous limiting dilution method in RD cell culture (RD). Virus-induced cytopathic effect (CPE) was assessed visually by examination of microplates under an inverted microscope. Viral infectious titers were calculated according to the Reed and Muench method (Reed and Muench) [28].

### 4.11. MTT Cytotoxicity Assay

The cytotoxicity of the synthesized coordination compounds was evaluated in human peripheral blood mononuclear cells (PBMCs; referred to as MNC), as well as in MDCK (Madin–Darby canine kidney) and RD lines.

Cell viability was assessed using the MTT assay [29,30], based on the ability of metabolically active cells to reduce the soluble tetrazolium salt 3-(4,5-dimethylthiazol-2-yl)-2,5-diphenyltetrazolium bromide (MTT) into insoluble intracellular formazan crystals.

Cell passaging, seeding, and cytotoxicity evaluation were performed according to internal laboratory protocols. Cells were seeded at a density of 2.5 × 10^5^ cells/mL and incubated for 24 h at 37 °C in a humidified atmosphere containing 5% CO_2_. After removal of the growth medium, 200 µL of DMEM containing various concentrations of the test compounds were added. Cells cultured in incomplete DMEM served as the negative control.

After 72 h, the medium was replaced, 50 µL of MTT working solution was added, and incubation was continued for 4 h. Subsequently, the supernatant was removed and the formazan crystals were dissolved in 100 µL of DMSO. Optical density (OD) was measured using a Tecan Sunrise RC.4 (Austria) at 540/620 nm.

The arithmetic mean OD value (Ȳ) for the negative control was calculated as:

(3) $$Y=\;\frac{y_{1}+\cdots +y_{n}}{n}=\;\frac{1}{n}\sum_{i=1}^{n}y_{i},$$

where *y_i_* is the OD value for each replicate and *n* is the number of replicates.

The percentage of viable cells for each concentration was calculated as:

(4) $$Cell\;viability\;\left(\%\right)=\;\frac{Y_{i}}{{\overset{-}{Y}}_{NC}}\;\times 100\%,$$

where *Y_i_* is the OD value for the test sample and Ȳ_NC_ is the mean OD of the negative control.

The half-maximal cytotoxic concentration (CC_50_), defined as the concentration causing 50% cell death, was calculated using linear interpolation:

(5) $${CC}_{50}=\left[\frac{X_{1}-50}{X_{1}-X_{2}}\times (M_{x2}-M_{x1})\right]+M_{x1},$$

where *X*_1_ and *X*_2_ represent percentages of viable cells above and below 50%, respectively; *M_x_*_1_ and *M_x_*_2_ correspond to the concentrations associated with *X*_1_ and *X*_2_.

Peripheral blood mononuclear cells were isolated by density gradient centrifugation using Histopaque (Sigma-Aldrich, Burlington, MA, USA; ρ = 1.077 g/mL) at 3000 rpm for 20 min at 4 °C. Cells were resuspended in RPMI-1640 medium (Sigma-Aldrich, Burlington, MA, USA). Cell viability was assessed by trypan blue exclusion; only cell preparations with viability > 90% were used in experiments.

PBMCs were cultured in complete RPMI-1640 medium supplemented with 10% fetal bovine serum (FBS), 2% L-glutamine, and 1% antibiotic–antimycotic solution in a CO_2_ incubator (37 °C, 5% CO_2_, 95% humidity).

Cell counting was performed using a Goryaev hemocytometer with trypan blue staining. The percentage of viable cells was calculated as:

(6) $$Viable\;cells\left(\%\right)=\frac{N_{1}\times 100\%}{N},$$

where *N* is the total number of cells counted in five large squares and *N*_1_ is the number of unstained (viable) cells.

Cell concentration was calculated as:

(7) $$C=\frac{N}{N_{1}}\times 225\times 1111\times 2,$$

where *C* is the number of cells per mL; 225 is the chamber conversion factor; 1111 is the Goryaev chamber coefficient; and 2 is the dilution factor.

For compound KC-248, the tested concentration range was 0.02–5.0 mg/mL, whereas for KC-246 it was 0.002–0.5 mg/mL. DMSO (0.1%) was used as the solvent for KC-246. Incubation was carried out for 48 h, after which cytotoxicity was determined using the MTT assay as described above.

## 5. Conclusions

Quantum chemical calculations confirmed the structural feasibility of lithium iodide coordination complexes with sodium sulfadimidine and gentamicin sulfate, supporting the proposed coordination-driven assembly. Based on these findings, two new active pharmaceutical substances (APSs), KC-246 and KC-248, were successfully synthesized and physicochemically characterized. Their key quality attributes (pH, melting point, iodine/iodide content) remained within acceptable limits over a 3-month period. The FT-IR analysis of KC-246 and KC-248 further confirms preservation of the parent molecular frameworks (sulfathimidine and gentamicin sulfate, respectively), while the appearance of additional bands at ~1011 cm^−1^ and ~783 cm^−1^, assigned to Li–O stretching vibrations, provides direct evidence of lithium coordination and successful formation of the modified complexes. UV-Vis spectroscopy revealed π-π* and n-π* transitions, indicating the presence of aromatic systems, carbonyl groups, and heteroatoms (O, N), consistent with coordination complex formation.

In vitro safety evaluation in PBMC cultures demonstrated that KC-248 exhibited lower cytotoxicity (CC_50_ = 0.425 mg/mL; non-toxic concentration 0.106 mg/mL) compared to KC-246 (CC_50_ = 0.179 mg/mL; non-toxic concentration 0.045 mg/mL). Both compounds showed broad-spectrum antimicrobial activity against Gram-positive, Gram-negative bacteria, and yeast-like fungi. KC-246 demonstrated higher potency, with minimum bactericidal concentrations ranging from 0.794 to 0.198 μg/mL, whereas KC-248 exhibited activity in the range of 2.093 to 0.523 μg/mL.

Antiviral studies confirmed activity against influenza A/Swine/Iowa/15/30 (H1N1) and herpes simplex virus type 1. KC-246 was active at lower concentrations (0.365–0.183 mg/mL for H1N1; 0.330–0.165 mg/mL for HSV-1) compared to KC-248 (1.275 and 1.300 mg/mL, respectively), indicating higher antiviral potency.

Overall, KC-246 emerges as the more active compound, combining strong antimicrobial and antiviral activity, while KC-248 demonstrates a more favorable cytotoxicity profile. Studies on excipient compatibility and dosage form development are ongoing.

Despite promising results, this study has several limitations. Although FT-IR analysis confirmed key structural features of the synthesized iodine-based coordination compounds, full characterization using NMR, MS, and elemental analysis is still ongoing. In addition, the biological evaluation was mainly limited to in vitro studies, and further in vivo investigations are required to confirm long-term safety and therapeutic potential. Finally, while theoretical calculations supported the proposed mechanisms, additional mechanistic studies are needed for deeper understanding of molecular interactions. Future work will address these aspects to provide more comprehensive validation of the developed compounds.

## Figures and Tables

**Table 1:** Complexation energies (ΔE, kcal/mol) and coordination bond lengths (Å).

Structure	−∆E	Me-O	Me-N
a	72.39	1.93	2.12
b	84.44	2.00	2.10

**Table 2:** Complexation energy (ΔE, kcal/mol), I_2_ bond energy in the complex (ΔE_1_, kcal/mol), and coordination bond length (Å).

Structure	−∆E	−∆E^1^	Li-O	Li-I^1^	Li-I^2^	I^1^-I^2^	I^2^-I^3^
c	27.23	20.48	1.92	2.62	2.96	3.07	2.91

**Table 3:** Physicochemical characterization.

Compound Name	Solubility	pH	Melting Point, °C	Iodine, g/kg	Iodide, g/kg
				Titration	
KC-246	Water, DMSO, acetone 1 g/10 mL (soluble)	2.35 ± 0.01	56–57 ± 0.71	101.67 ± 0.57	266.68 ± 0.82
KC-248	Water 1 g/5 mL (freely soluble)	2.35 ± 0.01	60–61 ± 1.0	100.78 ± 2.26	166.87 ± 1.29

**Table 4:** Quantification of iodide ions using CE.

Indicator	KC-246	KC-248
Iodide concentration, mg/L	382.99 ± 0.76	238.80 ± 0.62
Peak area, S (mEA × s)	1008.97	629.13

**Table 5:** Quantification of lithium cations using CE.

Indicator	KC-246	KC-248
Cation concentration, mg/L	11.43 ± 0.11	9.15 ± 0.04
Peak area, S (mEA × s)	14.005	11.215

**Table 6:** Physicochemical characterization after 3-month storage.

Compound Name	Solubility	pH	Melting Point, °C	Iodine, g/kg	Iodide, g/kg
				Titration	
KC-246	Water, DMSO, acetone 1 g/10 mL (soluble)	2.31 ± 0.02	57–58 ± 0.58	99.78 ± 1.05	286.95 ± 0.98
KC-248	Water 1 g/5 mL (freely soluble)	3.92 ± 0.05	62–64 ± 0.25	99.47 ± 2.13	168.17 ± 1.97

**Table 7:** Spectral characteristics of the synthesized complex compounds (UV spectra).

Compound Name	λ_max_, nm	A_max_	Transitions
KC-246	194.06	1.63	σ → σ*
	225.57	1.16	*n* → π*
	292.40	0.53	*n* → π*
	358.35	0.34	*n* → π*
Sodium sulfadimidine	194.06	1.73	*n* → π*
	240.91	0.99	*n* → π*
	259.09	1.01	*n* → π* or π → π*
KC-248	194.18	1.91	σ → σ*
	225.13	1.46	π → π*
	293.00	0.27	*n* → π*
Gentamicin sulfate	-	-	-

**Table 8:** Percentage of viable PBMCs after 48 h exposure to KC-246 and KC-248 substances.

Concentrations, mg/mL	KC-246 M ± SD (n = 4)	Concentrations, mg/mL	KC-248 M ± SD (n = 4)
0.50	28.7 ± 2.6	5.00	18.0 ± 4.2
0.25	50.9 ± 2.3	2.50	18.7 ± 1.3
0.125	87.4 ± 7.9	1.25	22.7 ± 4.5
0.063	84.0 ± 8.8	0.62	21.8 ± 1.9
0.031	98.5 ± 8.6	0.31	22.2 ± 1.6
0.016	97.8 ± 10.7	0.15	56.7 ± 7.7
0.008	99.3 ± 3.4	0.08	63.9 ± 2.8
0.004	102.2 ± 10.9	0.04	78.9 ± 8.5
0.002	96.4 ± 14.7	0.02	122.5 ± 15.1
CC_50_, mg/mL	0.179	CC_50_, mg/mL	0.072

**Table 9:** Assessment of the cytotoxic effect of the new compounds KC-246 and KC-248 in MDCK and RD cell culture models after 72 h of incubation.

Compound Name	CC_50_, on MDCK Cell Celture, (mg/mL)	CC_50_, on RD Cell Culture, (mg/mL)
KC-246	0.730 ± 0.06	0.660 ± 0.3
KC-248	2.550 ± 3.30	2.600 ± 1.60

**Table 10:** Results of the antimicrobial activity of KC-246 and KC-248 substances.

Test Strains	Compound Name	
	KC-246	KC-248
	Minimum Bactericidal Concentration (MBC) Values, μg/mL, Expressed as I_2_	
*S. aureus* ATCC 6538	0.198	0.787
*S. aureus* ATCC 33591	0.397	0.393
*E. coli* ATCC 8739	0.794	0.787
*E. coli* ATCC 2523	0.794	0.787
*S. enterica* ATCC 14028	0.794	3.149
*S. enterica* ATCC 35988	0.397	0.787
*Kl. pneumoniae* ATCC 10031	0.794	0.787
*Kl. pneumoniae* ATCC 2524	0.794	0.393
*P. aeruginosa* ATCC 9027	0.397	1.574
*P. aeruginosa* TA2	0.794	3.149
*Ent. hirae* ATCC 10541	0.794	0.787
*Ent. faecalis* ATCC 51575	0.397	0.787
*C. albicans* ATCC 10231	0.794	3.149
*C. albicans* SCAID PHPX 1-2019	0.794	3.149

**Table 11:** Assessment of the virus-inhibitory activity of KC-246 and KC-248 substances on the influenza A/Swine/Iowa/15/30 (H1N1) virus model.

Compound Name	Concentration, mg/mL	K_u_, %
KC-246	0.365	100
	0.183	100
	0.091	21.7
KC-248	1.275	100.0
	0.638	66.7
	0.319	4.5

**Table 12:** Assessment of the virus-inhibitory activity of KC-246 and KC-248 substances on the herpes simplex virus type 1, strain “Victory” model.

Compound Name	Concentration, mg/mL	Ku, %
KC-246	0.330	100
	0.165	100
	0.083	33.3
KC-248	1.300	100
	0.650	4.8
	0.325	0

## Data Availability

The original contributions presented in the study are included in the article and further inquiries can be directed to the corresponding author/s.

## Abbreviations

The following abbreviations are used in this manuscript:

APS	Active Pharmaceutical Substance
AMR	Antimicrobial Resistance
ATCC	American Type Culture Collection
BGE	Background Electrolyte
CC_50_	Half-maximal Cytotoxic Concentration
CDC	Centers for Disease Control and Prevention
CE	Capillary Electrophoresis
CLSI	Clinical and Laboratory Standards Institute
CPE	Cytopathic Effect
CRS	Certified Reference Standard
DAD	Diode-Array Detector
DFT	Density Functional Theory
DMSO	Dimethyl Sulfoxide
FBS	Fetal Bovine Serum
GLP	Good Laboratory Practice
GMP	Good Manufacturing Practice
HSV-1	Herpes Simplex Virus Type 1
MBC	Minimum Bactericidal Concentration
MDCK	Madin–Darby Canine Kidney
MES	Ministry of Education and Science
MTT	3-(4,5-dimethylthiazol-2-yl)-2,5-diphenyltetrazolium bromide
OD	Optical Density
PBMC	Peripheral Blood Mononuclear Cell
RD	Rhabdomyosarcoma
RT	Room Temperature
UV-Vis	Ultraviolet–Visible (spectroscopy)
